# Exploration of Safety, Pharmacokinetics and Selected Pharmacodynamic Mechanisms of High-Dose Se-Methylselenocysteine, L-Selenomethionine and Selenite in a Phase Ib Trial in Cancer Patients

**DOI:** 10.3390/ijms27156618

**Published:** 2026-07-24

**Authors:** Catherine M. Turnbull, Stephen O. Evans, Linda M. Peters, Renee J. Goodall, Craig K. Burns, Hugh J. B. Goodman, Natalie Briggs, Michael B. Jameson

**Affiliations:** 1 Faculty of Medical and Health Sciences, Waikato Clinical Campus, University of Auckland, Hamilton 3204, New Zealand; cat.turnbull@auckland.ac.nz; 2 School of Pharmacy and Biomedical Sciences, University of Waikato, Hamilton 3240, New Zealand; 3 School of Science, University of Waikato, Hamilton 3240, New Zealand; 4 Haematology Department, Waikato Hospital, Hamilton 3204, New Zealand; 5 Oncology Department, Waikato Hospital, Hamilton 3204, New Zealand

**Keywords:** selenium, pharmacokinetics, methylselenocysteine, selenite, selenomethionine, cancer, clinical trial, selenoprotein P, genotoxicity, glutathione

## Abstract

High-dose selenium (Se) as selenomethionine (SLM) and sodium selenite (SS) can improve the efficacy of cancer therapies while reducing toxicity. Se-methylselenocysteine (MSC) is effective in preclinical models but has not been evaluated in cancer trials. This phase Ib randomised, double-blinded trial explored the safety, pharmacokinetics (PK) and selected pharmacodynamic (PD) mechanisms of MSC, SLM and SS to guide future trials. Nine participants with metastatic cancer took Se 1600 μg/day for 4 weeks, then 6400 μg/day for 4 weeks, as MSC, SLM, or SS, with safety, PK, and PD assessments at baseline then every 4 weeks for 12 weeks. No dose-limiting toxicities occurred, and all adverse events were grades 1–2, mainly gastrointestinal. Total plasma Se increased after 8 weeks to mean 27.2 μM with SLM, 4.14 μM with MSC and 3.90 μM with SS. No significant changes in DNA damage or intracellular glutathione were observed in peripheral blood mononuclear cells. Mean plasma selenoprotein P (SEPP) increased from 3.74 mg/L at baseline to 4.66 mg/L and 5.13 mg/L after 4 and 8 weeks (*p* = 0.136 and 0.104, respectively). More detailed evaluations of safety, PK and PD mechanisms are needed to determine a recommended Se compound and dose for larger trials in combination with cancer therapies.

## 1. Introduction

Selenium (Se) is an essential trace element with multiple roles critical to human health, including in antioxidant pathways, redox signalling, DNA stability, thyroid hormone metabolism, protein and nucleic acid production, immune function, and fertility [1]. It is perhaps best known for its role in cancer prevention [2], which may contribute to the widespread use of Se as a nutritional supplement after a cancer diagnosis [3]. However, another important role for Se lies in its potential to improve cancer outcomes through modulating the efficacy and toxicity of cancer treatments [4].

Most cancer treatments, including chemotherapy, radiation and molecularly targeted drugs, cause significant toxicity to normal cells at therapeutic doses for cancers, resulting in a so-called “narrow therapeutic window” [5,6]. There is robust preclinical evidence that Se compounds can often widen this therapeutic window by reducing normal tissue toxicities without compromising the efficacy of these cancer treatments and may, in some circumstances, enhance them [4]. If this were confirmed in definitive clinical trials, then it would be a significant advance and could be widely and affordably implemented in clinical practice.

Despite numerous clinical trials using Se compounds with various cancer treatments, it is not yet clear whether Se has a useful role to play for cancer patients, with some trials demonstrating evidence of benefit and others not [4,7]. The clinical trials conducted to date, including randomised controlled trials (RCTs), are mostly underpowered, heterogeneous, and conducted in populations with varied baseline Se status, using different Se compounds, doses, schedules and routes of administration, and with differing cancer treatments and varying outcome measures [4,7,8]. They are also based on a number of differing hypotheses, variably including aims such as optimising selenoprotein function (including antioxidant activity) in populations with inadequate Se status, generating reactive oxygen species and related cytotoxicity, increasing methylselenol production, achieving plasma concentrations informed by preclinical studies, and targeting pharmacodynamic pathways where Se differentially mediates normal and malignant cell toxicity and death in response to cancer treatment [4].

A more effective and efficient approach to determining the role of combining Se compounds with cancer treatments could be to more comprehensively adapt principles used for anticancer drug development, such as incorporating early biomarkers for efficacy and safety evaluation and evaluation of multiple dose levels in late-phase development [5]. Many of the mechanistic insights and most-effective dosing strategies for different Se compounds identified in preclinical studies have not been implemented or adequately evaluated in clinical trials [4]. This includes assessing the effect of Se compounds on the pharmacodynamic (PD) mechanisms that mediate the effects of different Se compounds in this setting and the pharmacokinetic–pharmacodynamic (PK-PD) relationship. Similarly, while trials report the acute toxicities of Se compounds alone or in combination with cancer treatments, few have evaluated toxicities that can have late adverse consequences, such as genotoxicity increasing the risk of second malignancies, particularly in combination with DNA-damaging cancer treatments [9,10,11,12]. The compelling preclinical data clearly justifies conducting further well-designed clinical trials that are informed by this broad evidence base and that integrate correlative-translational endpoints along with clinical outcomes both in single-arm trials and RCTs [4].

We previously reported a randomised phase Ib clinical trial that administered three Se compounds (L-selenomethionine [SLM], sodium selenite [SS] and Se-methylselenocysteine [MSC]) dosed orally at Se 400 μg/day for 8 weeks to 24 participants with solid malignancies or chronic lymphocytic leukaemia [13]. SLM and SS were selected because they have been most commonly evaluated in previous trials, and MSC was included because of its superior efficacy compared to the other two compounds in some preclinical studies [14,15] and its greater efficiency in generating methylselenol, a key metabolite that mediates many effects of Se on cancer prevention and interactions with cancer treatments [16,17,18,19]. We found no genotoxicity or clinically concerning toxicity from any Se compound and no impact on PD pathways involving endoplasmic reticulum (ER) stress and angiogenesis in benign or malignant peripheral blood mononuclear cells (PBMCs) in the solid tumour and chronic lymphocytic leukaemia (CLL) groups, respectively [13,20].

This outcome was anticipated from preclinical studies, as Se 400 μg/day exceeds the dose needed to maximise selenoprotein function in populations with inadequate Se intake but is insufficient to elicit significant effects in several key PD pathways [12,20]. However, we considered it important to evaluate safety and the PK-PD response at that dose before continuing trial recruitment at the higher dose levels reported in this second phase of the trial. Due to funding and time constraints, we elected not to recruit further CLL patients and instead focused on gathering safety and pharmacokinetics (PK) data on the Se compounds at the higher dose levels, which was not previously available for MSC. The trial protocol was amended to include intra-participant dose escalation to reduce the number of participants required. We then randomised this second cohort of nine cancer patients to take one of the three Se compounds orally at Se 1600 μg/day of elemental Se for four weeks then Se 6400 μg/day for four weeks. We report here the clinical and laboratory safety (including DNA damage in PBMCs), total plasma Se PK, intracellular glutathione in PBMCs and, additionally for both cohorts, plasma selenoprotein P (SEPP) concentrations.

## 2. Results

Given the small cohort size (*n* = 3 per arm), all statistical analyses should be considered exploratory. Statistical interpretation is variable in these conditions, and *p*-values should be read with caution. Consequently, the results are intended to be preliminary rather than confirmatory.

### 2.1. Recruitment

**Figure 1:**
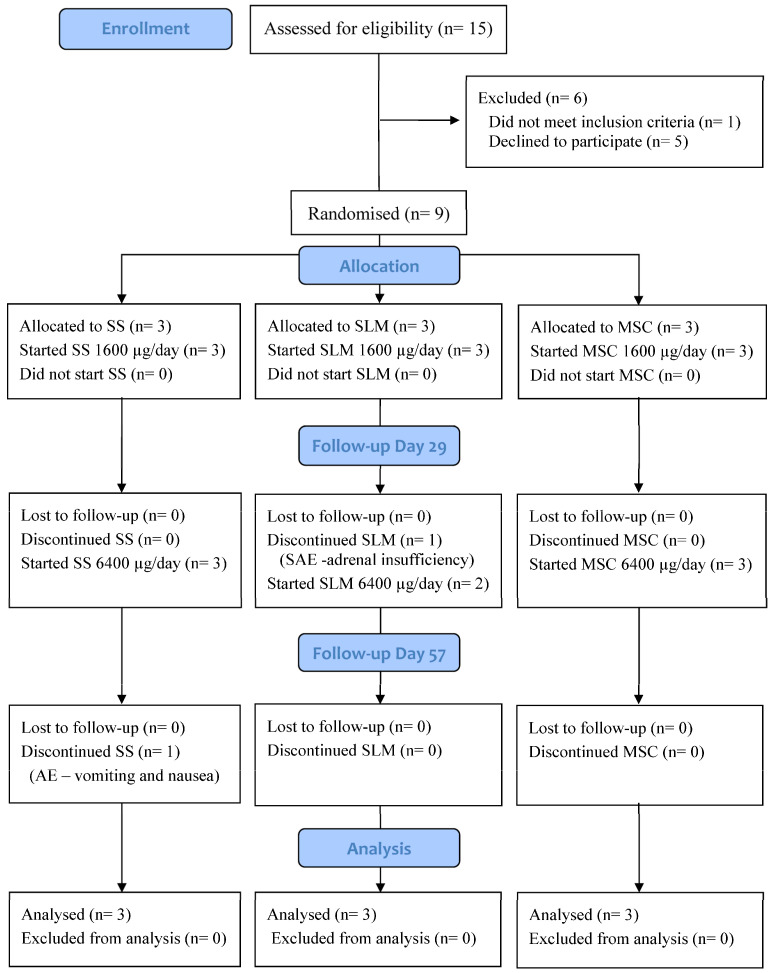
CONSORT flow diagram.

Between June 2023 and March 2024, nine participants were recruited and randomised across three treatment arms (Figure 1). Baseline participant characteristics were comparable across the arms (Table 1).

### 2.2. Safety Results

Differences in adverse events (AEs) patterns across the three compound groups are only evaluated descriptively due to the small cohort size. All three Se compounds were generally tolerated well, with no dose-limiting toxicity (DLT) observed in this small cohort, but this trial is unable to compare safety profiles between compounds. Of the nine randomised participants, seven completed the 56-day treatment schedule. One participant discontinued SS on Day 37 (eight days after starting 6400 µg/day) due to grade 2 nausea and vomiting; no anti-nausea medication was prescribed. Another participant discontinued SLM on Day 29 (and did not start 6400 µg/day) when admitted to the hospital with symptoms including confusion, dizziness, fatigue, and anorexia. These were subsequently attributed to acute adrenal insufficiency from pembrolizumab and resolved with corticosteroid replacement; this sole serious adverse event (SAE) was considered to be unrelated to the Se treatment, though a possible interaction with SLM cannot be discounted.

All treatment-emergent AEs across the MSC, SS, and SLM groups were grade ≤2 (Table 2). The most common were gastrointestinal symptoms (diarrhoea, nausea, vomiting), attributed to all three Se compounds at both dose levels. At 1600 µg/day, single-occurrence AEs included hypophosphataemia, urinary frequency, foot pain, bloating, and headache. At 6400 µg/day, grade 1 and 2 nausea and grade 2 vomiting occurred; other grade 1 AEs included bloating, abdominal cramping, diarrhoea, and rash. Most AEs were of brief duration and/or intermittent.

### 2.3. Pharmacokinetic Results

**Figure 2:**
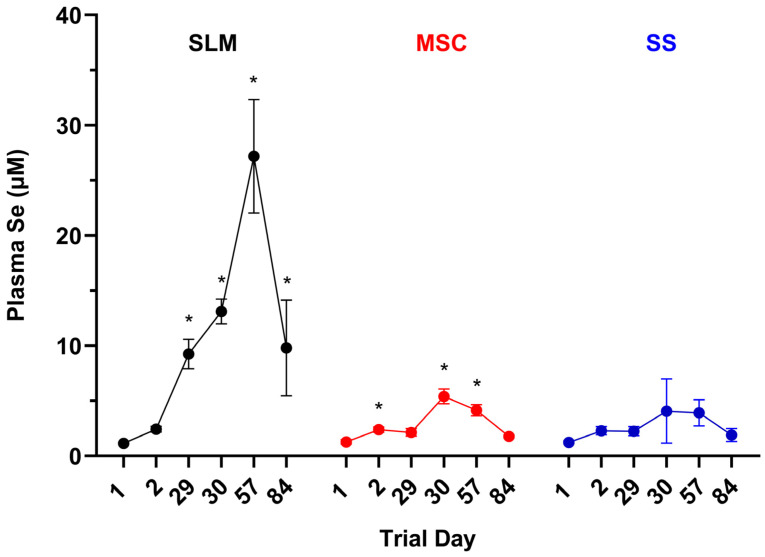
Total plasma Se mean (±SD) trough concentrations measured over 56 days of treatment (1600 µg/day Days 1–28, then 6400 µg/day Days 29–56) and 4 weeks after the last dose (Day 84). * *p* < 0.05 compared to Day 1 after adjustments for multiple comparisons within each Se compound group.

Total plasma Se concentrations were similar between groups at baseline (*p* = 0.65), then approximately doubled one day after the first dose of 1600 µg in all Se groups (Figure 2 and Table 3). Thereafter, the total plasma Se concentration response to SLM dosing was greater than that with either MSC or SS. In the SLM group, Se plasma concentrations rose progressively up to Day 57, reaching a mean of 27.2 µM, then reduced at Day 84 to 9.79 µM, still significantly higher than baseline (*p* = 0.016). By contrast, the increments in total plasma Se concentrations with MSC or SS were much smaller at each dose level (Figure 2 and Table 3), and Day 84 total plasma Se concentrations were not significantly different from baseline (*p* = 0.48 and *p* = 0.95, respectively).

### 2.4. DNA Damage Assay

**Figure 3:**
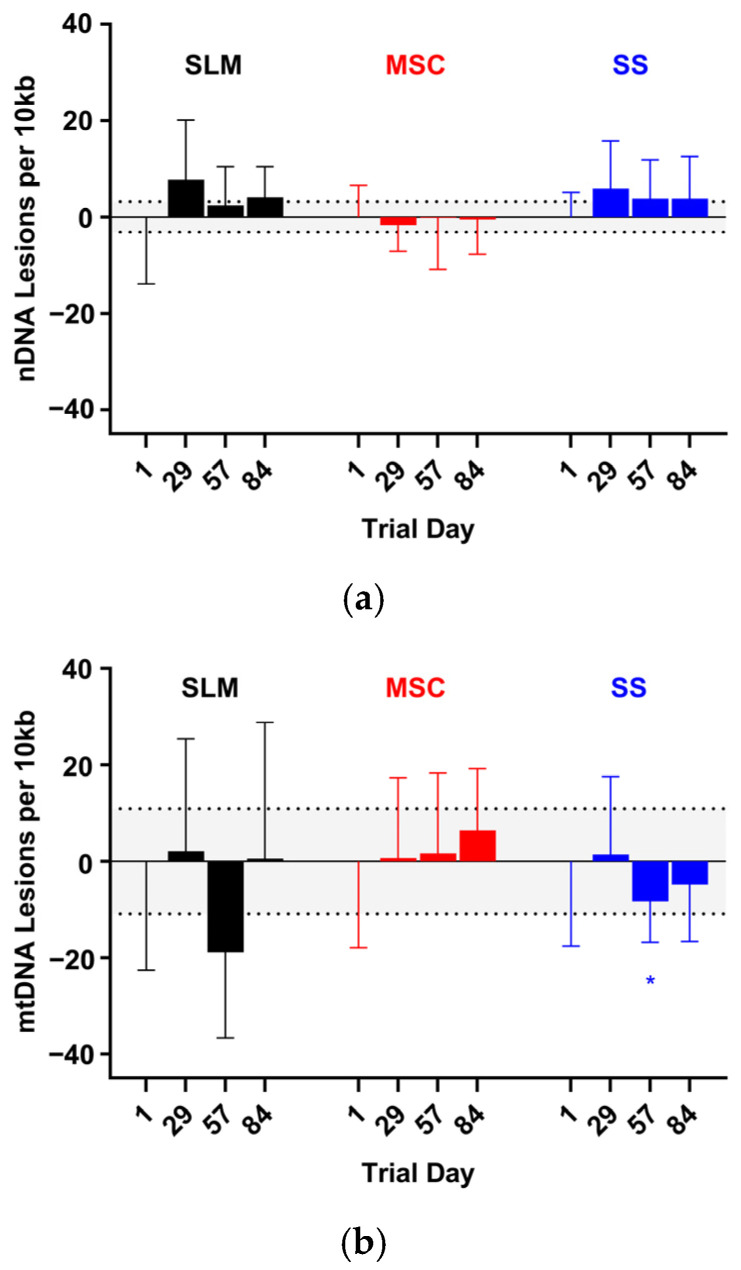
DNA damage: the mean (±SD) changes in DNA lesion rates per 10 kilobases (kb) in (**a**) nDNA and (**b**) mtDNA relative to pretreatment baseline in each group on Days 1, 29, 57, and 84 in each Se compound group. The grey horizontal bars represent the 95% confidence interval for within-participant variance in DNA lesion rates across two pretreatment samples taken 1–2 weeks apart from all participants. * *p* < 0.05 after multiple comparisons tests within each Se compound group.

The genotoxicity of the three compounds was assessed in PBMCs over the short observation period of the trial using DNA lesion rates in nuclear DNA (nDNA) (Table 4) and mitochondrial DNA (mtDNA) (Table 5). Lesion rates were quantified relative to the participant’s pretreatment baseline, and 95% confidence intervals were derived from two pretreatment samples collected from all participants 1–2 weeks apart. Overall, no statistically significant changes were observed in nDNA lesion rates over time (*p* = 0.270) or by Se compound (*p* = 0.473; Figure 3a). However, in the SS group, the Day 57 value was significantly lower than that at the Day 1 baseline after adjustment for multiple testing (*p* = 0.045). Similarly, mtDNA lesion rates did not differ significantly overall between treatments over time (*p* = 0.056) or by Se compound (*p* = 0.827; Figure 3b). These results indicate that none of the Se compounds induced detectable DNA damage in either mitochondrial or nuclear genomes under the conditions tested.

### 2.5. Selenoprotein P (SEPP)

**Figure 4:**
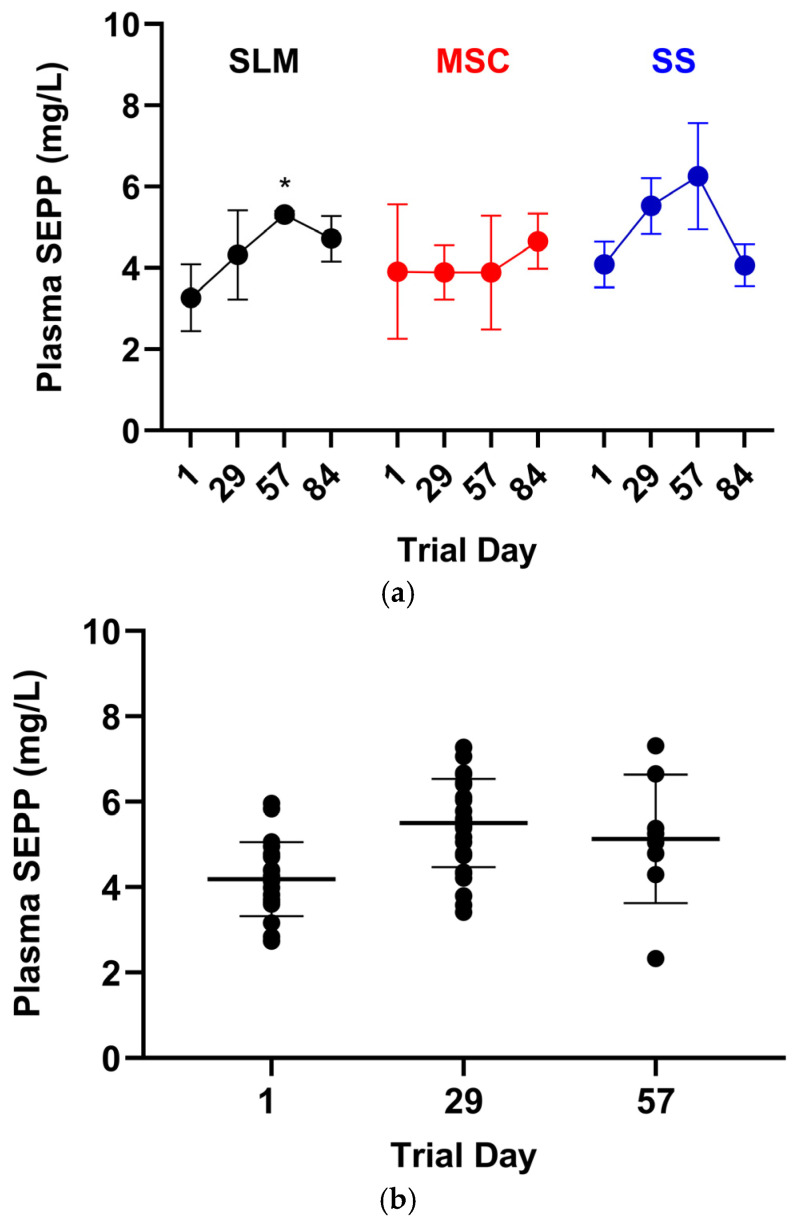
(**a**) Mean plasma (±SD) SEPP concentrations measured over 56 days of treatment and 4 weeks after the last dose (Day 84) in the second trial cohort. * *p* < 0.05 after correction for multiple comparisons within each Se compound group. (**b**) Plasma SEPP concentrations in all participants (n = 33) from both cohorts (Days 1 and 29 in both cohorts, Day 57 in the second cohort only); lines show the mean and SD.

The SEPP values in the SLM group in the first cohort were corrected for the non-specific incorporation of SLM into SEPP, which was estimated to account for approximately 45% of the increase in SEPP concentration in that group [21]. No significant differences were seen in the mean (±SD) plasma SEPP concentration at baseline between the Se compound groups in the second cohort (*p* = 0.595; Figure 4a) or between the first and second cohorts (*p* = 0.119). In the second cohort, the increase in mean plasma SEPP from 3.74 (0.91) mg/L at baseline to 4.66 (1.04) mg/L at Day 29 and 5.13 (1.50) mg/L at Day 57 was not significant (*p* = 0.136 and 0.104, respectively). However, for both cohorts combined (Figure 4b), the increase in mean plasma SEPP from 4.19 (0.86) mg/L at baseline to 5.50 (1.03) mg/L on Day 29 was statistically significant (*p* <0.001), without a significant difference between the increase in each cohort (means 1.45 and 0.93 mg/L in the first and second cohorts, respectively, *p* = 0.392). There was no significant increment in plasma SEPP concentration with increased Se intake from 1600 to 6400 µg/day from Days 29 to 57 in the second cohort (*p* = 0.855). While significant increases in plasma SEPP were seen in the first (400 µg/day) cohort with all three Se compound groups (Table 6), statistically significant changes in the second cohort were only seen in the SLM group between Day 1 and Day 57 (Table 7). The application of Oldham’s method to the SEPP data for all participants concluded that the increment in SEPP was not significantly different over the range of baseline values (correlation r = 0.24, 95% CI −0.09 to 0.52, *p* = 0.15; regression slope = 0.19, *p* = 0.15) [22].

### 2.6. Intracellular Glutathione (GSH)

**Figure 5:**
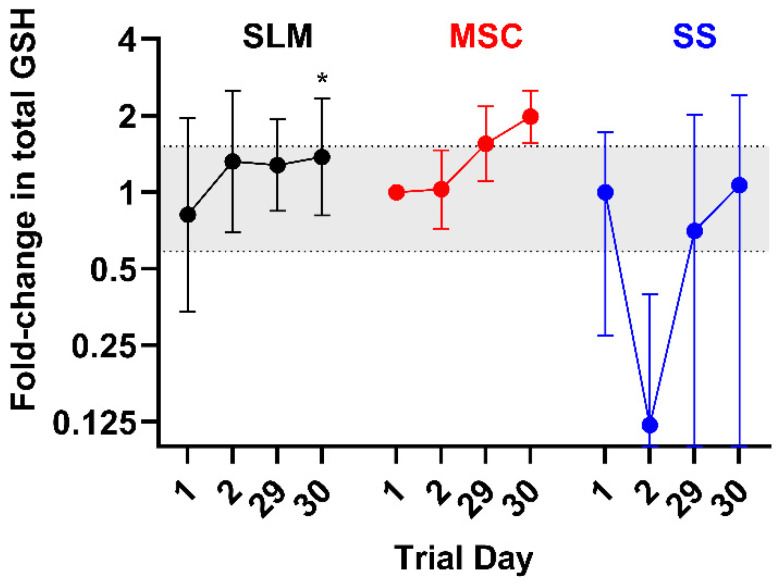
Mean (±SD) fold changes within each group in total intracellular glutathione (GSH) in peripheral blood mononuclear cells, normalised to total protein concentration, relative to the concentration of the first pretreatment sample. Samples were collected on Days 1 (prior to any Se), 2 (1 day after the first dose of 1600 µg Se), 29 (the last day of 1600 µg Se), and 30 (1 day after the first dose of 6400 µg Se). The grey horizontal bar represents the 95% CI for within-participant variance in fold change in total GSH across two pretreatment samples taken 1–2 weeks apart. * *p* < 0.05 after correction for multiple comparisons within each Se compound group.

The mean (±SD) total intracellular GSH at baseline was 0.001 (0.003) mg/mL when normalised with each participant’s total intracellular PBMC protein concentration. No significant differences were seen between groups in the baseline total GSH (*p* = 0.525) or in the variance between the two pretreatment samples (*p* = 0.552). Preclinical work showed that a change in GSH in PBMCs with Se was present over the first 24 h then had resolved by 48 h [12]. We, therefore, examined the change in GSH over the first 24 h at each dose level, i.e., on Days 1 and 2, then Days 29 and 30. The fold change in total GSH levels over time is shown in Table 8 and Figure 5. Neither mixed-effects analysis including all participants nor analysis by Se compound group revealed any significant differences in fold change in total GSH between Day 1 and any subsequent timepoints, or between Day 29 and Day 30, except for the difference between Day 1 and Day 30 in the SLM group, which remained significant after correction for multiple comparisons (*p* = 0.024).

## 3. Discussion

This report follows on from the reports on the 400 µg/day cohort in this phase 1b randomised double-blinded trial [13,20] and compares the same three Se compounds at the higher dose levels of 1600 µg/day and 6400 µg/day. While SLM and SS have been studied at much higher doses than those evaluated in this trial [23,24,25,26], this is the first clinical evaluation of MSC at repeated doses exceeding 800 µg/day [27]. This trial, therefore, provides important new data on the safety and PK of MSC compared to SLM and SS at higher doses and on intracellular glutathione and plasma SEPP responses.

The small number of cancer patients in this trial and the dose-escalation design preclude comparison of the safety of the Se compounds and doses studied. However, it is reassuring that all three compounds were generally well tolerated, with no treatment-emergent AEs greater than grade 2 reported. Only one of nine participants discontinued trial medication due to AEs attributed to Se (nausea and vomiting attributed to SS 6400 µg/day). The treatment-emergent AEs with all three Se compounds were clinical, without any significant biochemical, haematological or ECG AEs. The most common AEs were gastrointestinal (including nausea, vomiting, diarrhoea, and abdominal bloating), as well as fatigue and dizziness. Similar AEs have been reported with comparable higher doses of SS [28,29,30], SLM [24,25] and selenised yeast (predominantly SLM) [31].

The attribution of AEs should be interpreted cautiously, due to participants having metastatic cancer and some receiving concomitant therapies that are known to be associated with some of the reported AEs. Similarly, all participants received 1600 µg/day prior to the escalation to 6400 µg/day dosing, so the safety, tolerability, and PK of beginning at the higher dose cannot be determined from this trial. However, in the context of their intended use with patients receiving other cancer treatments, these Se compounds may be relatively tolerable when dosed at 1600 µg and 6400 µg of elemental Se per day.

The impact of Se compounds on DNA damage is complex, showing both DNA-protective and DNA-damaging properties. SS is reported to have significant genotoxicity even at low concentrations, compared to little-to-none reported with organic Se compounds such as SLM and MSC [10,32]. The long-run real-time polymerase chain reaction (LORD-Q PCR) assay used in this trial is more sensitive in detecting DNA damage than the Comet assay and additionally provides separate information on mtDNA and nDNA damage [33]. The higher background variation seen in damage rates in mtDNA than in nDNA in this trial has been reported by others [13,33,34]. However, none of the three Se compounds appeared to significantly impact existing rates of mtDNA or nDNA damage in PBMCs in this trial at any of the three dose levels and with the relatively short duration of exposure [13]. This is consistent with in vitro data showing no increase in DNA damage in the Comet assay when exposing PBMC to 5 µM methylseleninic acid (which is comparable to MSC in generating methylselenol and elimination) [12].

Caution should still prevail due to the possible long-term consequences of prolonged exposure to Se compounds, with reports variably describing deleterious or beneficial effects on DNA damage, including those induced by cancer treatments, which are both dose-dependent and Se compound-dependent [9,10,12,35]. The most compelling evidence for avoiding the long-term intake of Se >200 μg/day comes from a Danish placebo-controlled RCT supplementing older adults with selenised yeast 100, 200 or 300 µg/day for 5 years; whereas the lower two doses reduced mortality, the highest dose was associated with increased mortality rates that increased for 10 years after the trial intervention finished [36]. As participants were exposed to Se compounds for a short duration, the present trial cannot address the effects and risks of high-dose Se exposure.

The total plasma Se concentrations in the trial participants at baseline were 1.07–1.47 µM, considered adequate for the normal range in the NZ population (0.45–1.4 µM) but lower than the recommended optimal range for human health (1.6–2.0 μM) [1]. These concentrations increased much more with SLM than with MSC or SS, as expected [13,27]. The highest Se concentration with SLM was ~27 µM after dosing with 6400 µg/day for 4 weeks, compared to ~4 µM with MSC and SS. While total plasma Se returned to near-baseline levels 4 weeks after the completion of Se dosing with MSC and SS, after SLM, it was still seven-fold higher than baseline. The exaggerated increase in SLM in plasma compared to MSC or SLM is attributed to its non-specific incorporation into proteins, such as albumin, in place of methionine, which accounts for increased accumulation and slower clearance [37]. However, this does not appear to increase its in vivo activity compared to MSC in preclinical studies [14,19,38] or to affect its acute safety in this and other clinical trials [24,25,27].

Regression of individual participant data in this trial across the whole dose range (400–6400 µg/day) revealed that the increase in total plasma Se was linearly correlated with dose for each Se compound (r^2^ = 0.83, 0.97 and 0.89 for SS, SLM and MSC, respectively). However, the dose–response of plasma Se is highly variable between studies. For example, in two studies using SLM orally, the mean plasma Se concentration after SLM 9600 μg/day for 7 days was ~22 μM [39], compared to ~11 μM with SLM ~14,000 μg/day for 7 days [24]. The pharmacology of Se compounds is complex and highly variable, affecting all aspects (absorption, distribution, metabolism and elimination), with substantial inter-individual variability [40]. Therefore, it is important to integrate evaluation of both PK and PD endpoints along with efficacy and clinical outcomes in future trials [26].

Plasma SEPP increased significantly in response to Se dosing with all Se compounds, consistent with this New Zealand trial population having inadequate baseline Se status [41]. However, the increase in SEPP was similar across all Se compounds and in both the first dose cohort (400 μg/day, *n* = 23) and the second cohort (1600 then 6400 μg/day, *n* = 8). The mean and maximum concentrations achieved (5.5 mg/L and 7.3 mg/L, respectively) lie within the range (5–8 mg/L) reported in other studies in which SEPP concentrations plateau with Se supplementation, which is achieved in the nutritional range (up to 150 μg/day) [41,42]. The SEPP plateau range varies between populations and may be reached at different levels of Se intake [41,42]. For example, a US trial reported a baseline mean plasma SEPP of 7.3 mg/L with a mean plasma Se of 1.37 μΜ [27], in contrast to the baseline mean plasma SEPP of 4.2 mg/L with a mean total plasma Se of 1.22 μM in this trial. While mean plasma SEPP did not significantly increase in the US trial with SLM or MSC dosed orally at 400 μg/day or 800 μg/day for 84 days, indicating a Se-replete population, some subjects achieved SEPP levels >10 mg/L several hours after oral dosing on Day 1 or Day 84 [27]. In a European trial treating cancer or cardiac patients with daily IV SS at doses ranging from 1000 μg/day up to 34,000 μg/m^2^/day, about a third of patients achieved plasma SEPP >8 mg/L (maximum 14 mg/L) after 4–7 days [43]. However, the physiological or therapeutic significance of some individuals achieving these higher-than-expected plasma SEPP levels is uncertain. Overall, at supranutritional Se doses, plasma SEPP is no longer a useful biomarker for Se anticancer PD activity.

No significant change was seen in the total intracellular glutathione in PBMCs with any of the three Se compounds at any dose. In vitro data using 5 µM methylseleninic acid (which has an expected effect similar to MSC) showed a protective two-fold increase in GSH in PBMCs, similar to the trend seen in this trial with MSC 24 h after the first dose of 6400 μg, when plasma Se was 5.4 μM [12]. However, the GSH increase was not sustained in vitro beyond 24 h in that study or in this clinical trial, likely due to known homeostatic mechanisms [44].

The overall aim of this trial was to inform the conduct of future clinical trials using Se compounds to modulate the efficacy and toxicity of cancer treatments. While none of the participants had concurrent cytotoxic drugs or radiation, per protocol, five of nine were receiving the checkpoint inhibitor pembrolizumab, and two were taking exemestane, without any unexpected AEs being seen.

This phase Ib trial did not evaluate PD effects at the two higher dose levels in malignant cells and only evaluated plasma glutathione in PBMCs (with a response seen consistent with that reported in vitro). Therefore, the aim of evaluating the PK-PD relationship for the three Se compounds to inform the selection of Se compound and dose for use in future clinical trials has only partly been met. While no major safety signals were observed, the small cohort size and relatively short timeframe preclude drawing conclusions about safety over the longer duration expected with intervention trials.

Given preclinical evidence that MSC may have a more favourable efficacy and safety profile than SLM or SS when used with some chemotherapy drugs [4,14,19,38], this trial provides evidence to support MSC being taken forward for further evaluations in clinical trials. Within the limitations of the small cohort size and limited duration of exposure, there were no signals to suggest any safety concerns with MSC or SS across the doses used in this trial. The plasma levels of MSC achieved when participants were dosed at 6400 μg/day were comparable to those in vitro, at which beneficial interactions of methlyselenininc acid (another efficient methylselenol donor) were seen with cytotoxic drugs and radiation in PBMCs and in an acute leukaemia cell line [12].

Analysis of markers of angiogenesis and ER stress was not performed at the higher dose levels in this trial, as patients with CLL were not recruited, and these analyses are not expected to change significantly in PBMCs. Plasma vascular endothelial growth factor (VEGF) is not considered a reliable biomarker of angiogenesis or tumour treatment response [45]. However, collaborators are planning the analysis of DNA repair pathway gene expression in PBMCs and Se speciation in plasma and PBMCs.

Due to funding and time constraints, priority was given to completing safety assessment (including genotoxicity) and PK in the second trial cohort, which resulted in only nine participants informing safety and PK assessments, compared to 24 evaluated at 400 µg/day. Omission of a CLL cohort meant forgoing a comparison of PD responses between benign and malignant PBMCs, though the chances of seeing a significant difference were small, given the number of participants. We also introduced intra-participant dose escalation, which facilitated the evaluation of within-participant PK responses to Se dose increments, though with a 4-week duration of Se exposure at each dose (compared to 8 weeks in the lower dose cohort), but there was no evaluation of starting Se 6400 µg/day in Se-naïve participants.

This trial has provided evidence to support the inclusion of MSC with SLM and SS as Se compounds that could be evaluated in future trials in conjunction with cancer treatments. Whether MSC is more clinically effective than SLM or SS in reducing normal tissue toxicities from cancer treatments while increasing their efficacy, as demonstrated in some preclinical models [14,15], remains to be evaluated in further trials. These trials should incorporate more detailed evaluations of safety, PK and PD (including mechanisms mediating differential effects in normal and malignant cells) before determining a recommended Se compound and dose for further trials in combination with cancer therapies.

## 4. Materials and Methods

### 4.1. Patient Eligibility

Eligible participants had a pathologically confirmed diagnosis of metastatic solid cancer with no anticipated need for cytotoxic chemotherapy within the next 3 months. A decision was taken not to recruit any participants with CLL in this trial’s second phase due to time and funding constraints, accepting that this restricted PD evaluations to normal PBMCs and reduced the number of participants assessable for safety and PK at each dose level. The amended dose escalation design, however, enabled assessment of PK and PK-PD responses at two dose levels within each participant.

Eligibility criteria also required age >18 years old; an ECOG performance status of ≤2; adequate renal, liver, and bone marrow function; and a life expectancy of >6 months. Exclusion criteria included concurrent Se supplements >100 µg/day or treatment with cytotoxic chemotherapy, radiation therapy (RT) or systemic therapy that targets angiogenesis-signalling pathways in the prior 4 weeks. Treatment with any of these therapies was prohibited during the 12-week trial involvement, but established endocrine or immunotherapy treatment was permitted and continued during the trial. Ethical approval for the trial was granted by the Northern A Health and Disability Ethics Committee (13/NTA/172), and all participants provided written informed consent. The trial is registered with the Australian and New Zealand Clinical Trials Registry (ACTRN12613000118707).

### 4.2. Trial Design

This phase Ib dose-escalation, double-blinded trial randomised nine participants to one of the three arms (MSC, SLM or SS), with each arm balanced to include three participants. A computer-based permuted block randomisation (block size of three) and dispensing of blinded trial medication was managed by an external clinical trial pharmacist. Treatment assignment was concealed from participants, investigators, and research and laboratory personnel to maintain blinding. No placebo was used.

If trial treatment-related DLT was observed in two or more participants in a Se treatment arm, then recruitment into that arm would be terminated, but recruitment into arms involving other Se compounds could continue. DLT was defined as: absolute neutrophil count <1.0 × 10^9^/L lasting at least 7 days or associated with fever (>38.5 °C) or grade 3–4 sepsis; platelet count <50 × 10^9^/L lasting at least 7 days or accompanied by >grade 2 bleeding; clinically significant non-haematological toxicity >grade 3 despite appropriate treatment (including nausea or vomiting not controlled with appropriate therapy); grade 2 treatment-emergent neurotoxicity; and grade 2 allergic toxicity. Common terminology criteria for adverse events (CTCAE) version 4.0 was used for AE grading (https://dctd.cancer.gov/research/ctep-trials/for-sites/adverse-events, accessed on 19 July 2026).

### 4.3. Trial Treatment

Participants self-administered one Se capsule orally each day, which provided the equivalent of 1600 µg/day of elemental Se for 28 days, followed by Se 6400 µg/day for an additional 28 days. No modification of trial treatment was permitted other than a break of up to three days due to treatment intolerance, on two occasions. Sabinsa Corporation (20 Lake Drive, East Windsor, NJ 08520-5231, USA) provided all Se compounds as identical capsules. Pharmaceutical quality control test results were approved by Medsafe, Ministry of Health, New Zealand.

### 4.4. Trial Assessments

Safety assessments were performed at trial baseline and on Day 29 (end of 1600 µg/day dosing), Day 57 (end of 6400 µg/day dosing), and Day 84 (four weeks posttreatment). These included medical history, physical examination, urinalysis, ECG, and blood tests (CBC, renal/liver function, glucose, urate, calcium, phosphate, and coagulation). Participants maintained diaries of for recording adherence to selenium capsules, health and medication changes. For AE reporting, an AE persisting across multiple trial visits without documented resolution was considered a single event, with the highest grade experienced reported. Recurrence after resolution was recorded as a new event. The causality of AEs was investigator-assessed according to the trial protocol and classified in relation to the trial treatment.

Blood samples for PK, DNA damage and PD analyses were collected at each visit and additionally on Day 1, Day 2 and Day 30; processed to obtain plasma (1000× *g*, 10 min, 4 °C) and PBMCs; and stored at −80 °C as previously described [20]. The additional analyses enabled: (1) assessment of intra-participant PD variation prior to Se intervention, (2) trough Se levels 24 h after the first dose at each dose level, and (3) acute changes in selected PD responses 24 h after starting at each Se dose. Plasma and PBMC samples for analysis of Se speciation and DNA damage repair pathways were taken only on Days 1, 29 and 57 and will be reported elsewhere.

### 4.5. Plasma Se

Venous blood was collected in trace element tubes (K_2_-EDTA, BD), and total plasma Se concentrations were measured by ICP-MS (Agilent 7700; Agilent Technologies, CA, USA) at Canterbury Health Laboratories, Christchurch, New Zealand.

### 4.6. DNA Damage Assessment

DNA damage in nDNA and mtDNA was quantified using LORD-Q PCR, as previously described [33]. Each sample was tested four times. Lesion rates were calculated relative to DNA lesions in baseline samples. Four primer sets were designed to amplify short (<100 bp) and long (>3000 bp) fragments of nDNA (*p53* gene; NG_017013.2, positions 4197–7271 bp) and mtDNA (NC_012920, positions 11,492–15,214) (Appendix A).

### 4.7. Plasma Selenoprotein P (SEPP)

Plasma SEPP levels at Days 1, 29, 57, and 84 were measured using a commercial ELISA kit (SEB809Hu, Human Selenoprotein P1, Cloud-Clone Corp, Wuhan, China). Plasma was centrifuged at 1000× *g* for 2 min to remove residual fibrinogen and cell debris, then diluted 1:500 in PBS (pH 7.2).

Plasma SEPP was also analysed by LGC (Guildford, UK) in stored plasma samples taken on Day 1 and Day 29 from the first cohort dosed with MSC, SLM or SS at 400 µg/day [13,21].

### 4.8. Intracellular Glutathione (GSH)

An intracellular glutathione (GSH) assay was conducted on PBMCs collected from participants at baseline and on Days 1, 2, 29 and 30 to measure the total GSH (reduced and oxidised). PBMC lysates were assayed using the GSH/GSSG Ratio Detection Assay Kit II (ab205811, Abcam, UK), which is a thiol green-based fluorometric assay. PBMCs were lysed using Triton X-100 (Sigma-Aldrich, St. Louis, MO, USA) cell lysis buffer (with protease inhibitor cocktail); total protein concentration was quantified using the Bradford Assay (Bio-Rad, CA, USA). Protein samples were then deproteinised and neutralised using the TCA Deproteinising Sample Preparation Kit (ab204708, Abcam, UK) prior to the glutathione assay. Duplicates were run for all the samples and standards. The assay was run on SpectraMax M4 Microplate Reader (Molecular Devices, San Jose, CA, USA) (490 nm excitation, 520 nm emission). Assay results were normalised using the total protein content per sample and corrected for volume adjustments during the sample preparation.

### 4.9. Statistical Analysis

Statistical analyses were conducted using GraphPad Prism version 11.0 for Windows, GraphPad Software, Boston, MA, USA, www.graphpad.com. Significance was defined as two-sided *p* < 0.05. Baseline participant characteristics, safety, and toxicity were summarised through descriptive statistics. Baseline data were compared between Se compound groups using one-way ANOVA. Changes between baseline (or Day 1) and subsequent timepoints within each Se compound group for Se, SEPP and GSH assays were analysed using paired *t*-tests, then Se and GSH data for all participants in the second cohort (and SEPP data in each cohort) were analysed using two-way ANOVA (or mixed-effects modelling if missing data) with Dunnett’s, Tukey’s or Šídák’s multiple comparisons tests, as recommended. Given the limited sample size, this study was not powered for definitive statistical inference. Therefore, all statistical analyses were exploratory, and *p*-values should be interpreted with caution. The analyses were intended to support the descriptive evaluation of potential treatment-related trends rather than established statistically significant differences between groups. Analyses were performed using all available data, and tables note the number of samples/cases included in each analysis.

## Figures and Tables

**Table 1:** Participant characteristics.

Characteristics	MSC (*n* = 3)	SLM (*n* = 3)	SS (*n* = 3)
Mean age, years (range)	78 (76–80)	69 (62–73)	72 (66–78)
Sex			
Male	1	3	2
Female	2	0	1
ECOG PS *			
0	2	3	2
1	1	0	1
Malignancy			
Melanoma	2	1	2
Prostate cancer	0	1	1
Lung cancer	0	1	0
Breast cancer	1	0	0
Current Therapy			
Pembrolizumab	2	1	2
Exemestane	1	0	1
Nil	0	2	0

* Eastern Cooperative Oncology Group Performance Status.

**Table 2:** Treatment-emergent adverse events.

Adverse Events	MSC		SS		SLM	
	Grade		Grade		Grade	
	1	2	1	2	1	2
Se 1600 μg/day						
Diarrhoea	1	0	1	0	4 *	0
Nausea	1	0	0	0	0	0
Vomiting	1	0	0	0	0	1
Arthralgia	0	0	1	0	0	0
Headaches	0	0	1	0	0	0
Hypophosphatemia	0	1	0	0	0	0
Urinary Frequency	1	0	0	0	0	0
Confusion	0	0	0	0	0	1
Dizziness	0	0	0	0	1	1
Fatigue	0	0	1	0	0	1
Anorexia	0	0	0	0	0	1
Se 6400 μg/day						
Abdominal Pain	1	0	0	0	0	0
Bloating	0	0	1	0	0	0
Diarrhoea	0	0	2 **	0	0	0
Nausea	0	1	0	1	0	0
Vomiting	0	0	0	1	0	0
Maculopapular rash	0	0	1	0	0	0

* All 3 participants taking SLM reported at least one diarrhoea AE, in one of whom it recurred. ** Two participants taking SS reported an AE of diarrhoea.

**Table 3:** Mean total plasma Se (µM) concentrations (±SD) at each timepoint.

Timepoint	SLM, *n* = 3	*p* ^1^	MSC, *n* = 3	*p* ^1^	SS, *n* = 3	*p* ^1^
Day 1	1.13 (0.07)	-	1.27 (0.20)	-	1.22 (0.14)	-
Day 2	2.42 (0.26)	0.009	2.39 (0.24)	0.012 *	2.29 (0.38) ^2^	0.074
Day 29	9.25 (1.33)	0.010 *	2.14 (0.36)	0.089	2.24 (0.41)	0.024
Day 30	13.1 (1.12) ^2^	0.041 *	5.40 (0.66)	0.005 *	4.06 (2.91)	0.221
Day 57	27.2 (5.14) ^2^	0.088 *	4.14 (0.51) ^1^	0.057 *	3.90 (1.18) ^4^	0.161
Day 84	9.79 (4.34) ^3^	0.073 *	1.76 (0.13)	0.058	1.90 (0.60) ^2^	0.190

^1^ Statistical comparison was paired *t*-test against Day 1 values in each treatment arm. ^2^ *n* = 2. ^3^ One sample taken 4 weeks after the last Se dose (discontinued on Day 29, taking 1600 µg/day). ^4^ One sample taken at trial withdrawal on Day 37 (taking 6400 µg/day). * *p* < 0.05 after adjustment for multiple comparisons against Day 1 within each treatment arm.

**Table 4:** DNA damage assessed as mean change (±SD) in DNA lesion rate (per 10 kilobases) relative to the pretreatment baseline in nDNA.

Timepoint	SLM, *n* = 3	*p* ^1^	MSC, *n* = 3	*p* ^1^	SS, *n* = 3	*p* ^1^
Day 1	0.00 (13.9)	-	0.00 (6.62)	-	0.00 (5.13)	-
Day 29	7.73 (12.4)	0.835	−1.73 (5.35)	0.031	5.94 (9.85)	0.039
Day 57	2.42 (8.04)	0.191	−0.17 (10.7)	0.960	3.82 (8.07)	0.018
Day 84	4.09 (6.37)	0.324	−0.54 (7.18)	0.954	3.79 (8.76)	0.355

^1^ Statistical comparison was paired *t*-tests against Day 1 values in each group.

**Table 5:** DNA damage assessed as mean change (±SD) in DNA lesion rate (per 10 kilobases) relative to the pretreatment baseline in mtDNA.

Timepoint	SLM, *n* = 3	*p* ^1^	MSC, *n* = 3	*p* ^1^	SS, *n* = 3	*p* ^1^
Day 1	0.00 (22.6)	-	0.00 (18.0)	-	0.00 (17.6)	-
Day 29	2.07 (23.3)	0.411	0.66 (16.7)	0.548	1.422 (16.2)	0.796
Day 57	−18.9 (17.8)	0.600	1.66 (16.7)	0.365	−8.37 (8.47)	0.172 *
Day 84	0.60 (28.2)	0.926	6.42 (12.8)	0.089	−4.83 (11.8)	0.065

^1^ Statistical comparison was paired *t*-tests against Day 1 values in each group. * *p* < 0.05 on 2-way ANOVA or mixed effects analysis with Dunnett’s multiple comparison test within each treatment arm.

**Table 6:** Mean SEPP (±SD) concentrations (mg/L) of cohort 1 (400 µg/day) at Day 1 and Day 29.

Timepoint	SLM, *n* = 7	*p* ^1^	MSC, *n* = 8	*p* ^1^	SS, *n* = 8	*p* ^1^
Day 1	4.39 (0.99)	-	4.28 (0.7)	-	4.38 (0.84)	-
Day 29	6.33 (1.14)	0.001 *	5.60 (0.53)	<0.001 *	5.52 (0.77)	0.003 *

^1^ Statistical comparison was a paired *t*-test against Day 1 values in each treatment arm. * *p* < 0.05 after adjustment for multiple comparisons against Day 1 within each treatment arm.

**Table 7:** Mean SEPP (±SD) concentrations (mg/L) of cohort 2 (1600/6400 µg/day) at each timepoint.

Timepoint	SLM, *n* = 3	*p* ^1^	MSC, *n* = 3	*p* ^1^	SS, *n* = 3	*p* ^1^
Day 1	3.27 (0.82)	-	3.91 (1.65)	-	4.09 (0.56)	-
Day 29	4.32 (1.10)	0.403	3.89 (0.67)	0.992	5.26 (0.69)	0.182
Day 57	5.32 (0.09)	0.004 *	3.89 (1.40)	0.945	6.25 (1.31)	0.180
Day 84	4.72 (0.56)	0.182	4.66 (0.68)	^2^	4.06 (0.52)	0.975

^1^ Statistical comparison was a paired *t*-test against Day 1 values in each treatment arm. ^2^ *n* = 2. * *p* < 0.05 after adjustment for multiple comparisons against Day 1 within each treatment arm.

**Table 8:** Fold change in total intracellular GSH from the first pretreatment sample, normalised to total protein concentration in PBMCs.

Timepoint	SLM, *n* = 3	*p* ^1^	MSC, *n* = 2	*p* ^1^	SS, *n* = 3	*p* ^1^
Day 1	1.00 (0.61)	-	1.00 (0.02)	-	1.00 (0.73) ^2^	-
Day 2	1.53 (1.07)	0.639	1.06 (0.37)	0.854	0.12 (0.28) ^2^	0.222
Day 29	1.33 (0.54) ^2^	0.153	1.60 (0.53)	0.367	0.70 (1.31)	0.946
Day 30	1.47 (0.75) ^2^	0.017 *	2.01 (0.47)	0.196	1.07 (1.34)	0.184

^1^ Statistical comparison was a paired *t*-test against Day 1 values in each treatment arm. ^2^ *n* = 2. * *p* < 0.05 after adjustment for multiple comparisons against Day 1 within each treatment arm.

## Data Availability

The data described in this manuscript are available upon reasonable request. Access is granted for research projects that comply with legal and ethical standards. Data requests should be directed to Associate Professor Michael B. Jameson (michael.jameson@auckland.ac.nz).

## Abbreviations

The following abbreviations are used in this manuscript:

µg	Microgram
µM	Micromolar
AE	Adverse event
CI	Confidence interval
CLL	Chronic lymphocytic leukaemia
CTCAE	Common terminology criteria for adverse events
DLT	Dose-limiting toxicity
DNA	Deoxyribonucleic acid
ECG	Electrocardiogram
ER	Endoplasmic reticulum
GSH	Intracellular glutathione
IV	Intravenous
L	Litre
LORD-Q PCR	Long-run real-time polymerase chain reaction
m^2^	Metre squared
mg	Milligram
mL	Millilitre
MSC	Se-methylselenocysteine
mtDNA	Mitochondrial DNA
n=	Number of samples
nDNA	Nuclear DNA
NZ	New Zealand
PBMCs	Peripheral blood mononuclear cells
PD	Pharmacodynamics
PK	Pharmacokinetics
RCTs	Randomised controlled trials
RT	Radiation therapy
SAE	Serious adverse event
SD	Standard deviation
Se	Selenium
SEPP	Selenoprotein P
SLM	L-selenomethionine
SS	Sodium selenite
VEGF	Vascular endothelial growth factor

## Appendix A

### LORD-Q PCR Method

PCR reactions (10 µL) were run in duplicate on a Rotor-Gene 6000 (Qiagen, Hilden, Germany). For nDNA, reactions contained 1× buffer B1, 2.5 mM MgCl_2_, 0.5 U Hot FIREPol^®^ polymerase (Solis Biodyne, Tartu, Estonia), 200 µM dNTPs, 500 nM primers, 12.5 ng DNA template, and 5 µM SYTO82 (Thermo Fisher Scientific, Waltham, MA, USA). For mtDNA, 1× buffer B2 was used with the same concentrations, with an additional 0.5X S solution for short amplicons. Table A1 shows the primer sequences, annealing temperatures, and product sizes.

**Table A1:** Primers used for LORD-Q PCR to assess DNA damage in PBMC cells from patient samples and the annealing temperatures used.

Primer Name	Annealing Temp. (°C)	Sequence (5′-3′)	Product Size (bp)
*p53* forward	64.5	CATAACCGCAAATGGGAAAC	3075
*p53* long reverse		CGGGACGTGAAAGGTTAGAA	
*p53* short reverse	60	CGTCCTTTTGATGGCCTTT	45
Mitochondrial forward	63	CGCCTCACACTCATTCTCAA	3723
Mitochondrial long reverse		AATGTATGGGATGGCGGATA	
Mitochondrial short reverse	63	CAAGGAAGGGGTAGGCTATG	55

Cycling conditions (Table A2) included an initial denaturation (95 °C, 15 min), a hot start phase (10 cycles with 0.5 °C decrement per cycle), and amplification of 30 cycles (short) or 35 cycles (long), with extension at 72 °C for 240 s. Melt curves confirmed specificity.

**Table A2:** Cycling conditions of LORD-Q PCR.

Cycle Step	Incubation Times
Initial denaturation (1 cycle)	95 °C for 15 min
Hot start (10 cycles)	Step 1: 95 °C for 10 s
	Step 2: 65 °C (−0.5 °C/cycle) for 10 s
	Step 3: 72 °C for 240 s
Amplification (30–35 cycles)	Step 1: 95 °C for 15 s
	Step 2: 60–64.5 °C for 15 s
	Step 3: 72 °C for 240 s acquiring to cycling A (yellow channel)
	Step 4: 82 °C for 10 s acquiring to cycling B (yellow channel)
Melt curve	Ramp from 64 °C to 95 °C
	Hold for 90 s on the 1st step
	Hold for 5 s on the subsequent. Melt A (yellow channel)

Amplicon efficiencies were determined from standard curves, and mean Cp values from duplicate reactions were used to calculate lesion frequencies per 10 kb using Theorem A1.

Lesions per 10 kb = [(E_L_^Cpl(Sample)^ × E_s_^−Cps(Sample)^/E_L_^Cpl(Control)^ × E_s_^−Cps(Control)^)^1/a^ − 1] × 10,000

**Theorem** **A1.**
*DNA damage equation to calculate the DNA lesions per 10 kb. E_L_ and E_s_ are the average amplification efficiencies for the long and short products, respectively; C_p_ values are the threshold values determined by the Rotor-Gene Q software version 2.3.5.1 (C_pl_: long; C_ps_: short); and a is the number of base pairs of the long fragment.*
